# Scanning Auger Microscopy Studies of Silane Films Grown on Plasma-Modified HOPG Surfaces

**DOI:** 10.3390/polym11020307

**Published:** 2019-02-12

**Authors:** Jade K. Taylor, Jasmine R. Wiese, Sarah L. Harmer, Jamie S. Quinton

**Affiliations:** Flinders Institute for NanoScale Science and Technology, and Flinders Microscopy and Microanalysis, Flinders University, PO Box 2100, Adelaide, SA 5042, Australia; jade.taylor@flinders.edu.au (J.K.T.); Jasmine.Wiese@flinders.edu.au (J.R.W.); Sarah.Harmer@flinders.edu.au (S.L.H.)

**Keywords:** silane, PTMS, PDMMS, carbon, graphite, HOPG, plasma, XPS, SAM

## Abstract

The growth of silane films on plasma oxidized highly oriented pyrolytic graphite (HOPG) surfaces has been studied using wet chemical deposition of propyltrimethoxysilane (PTMS) and propyldimethylmethoxysilane (PDMMS). Scanning Auger microscopy (SAM) and X-ray photoelectron spectroscopy (XPS) were used to investigate the chemical composition and morphology of the silane films. The effects of several deposition parameters were examined, including the necessity of oxidation of the HOPG surface, addition of water with the silane, and rinsing before curing. The optimal conditions needed to create a complete uniform film differ for the two silanes due to differences in their structures. Both silanes require an oxidized HOPG surface for a film to grow, the addition of water with PTMS results in a thicker film, while the addition of water with PDMMS decreases the film growth. Rinsing of both samples before curing removes physisorbed species, leaving only the covalently bonded film on the surface.

## 1. Introduction 

The applications of silanes are extremely diverse, including corrosion protection films [1,2], coupling agents and cross linkers [1,2,3,4], and corner capping agents for nanoparticle molecules such as polyhedral oligomeric silsesquioxanes (POSS) [5]. In particular, applications of silane films on carbon substrates have been reported recently, including the formation of enzyme-based biosensors [6], stabilising agents for water-soluble graphene dispersions [7], modification of graphene properties [8], incorporation of graphene [9,10] or carbon nanotubes into a polymer matrix [3,4,11,12,13], polymer-electrolyte-membrane fuel cells [9], and the formation of silicon-based nanostructures [14].

Silane coupling agents (general formula R’Si(OR)_3_) are commonly used for providing adhesion between two dissimilar materials and have many properties that make them ideal for providing the chemical attachment in the applications listed above. These properties vary depending on the R’ functional group within the silane, which imparts a chemical functionality to the modified surface. Of particular interest is the formation of the silane film on the substrate surface, and it is for this reason that propyltrimethoxysilane (PTMS) and propyldimethylmethoxysilane (PDMMS) were chosen for this study on the formation of silane films on HOPG. While there have been a range of silanes used on carbon materials for various applications [6,7,8,9,10,11,14,15], for this study, these two simple model silanes were chosen. The inert propyl chain in these molecules minimizes intermolecular interactions as much as possible while still remaining stable in solution, allowing study of the growth mechanism with minimal interference from the functional group [16]. PTMS contains three hydrolysable groups, while PDMMS contains only one, resulting in a difference in the structure and growth mechanism between the two types of film. PDMMS can only form one chemical bond, and thus bind to one site, either to the substrate surface or to another PDMMS molecule, whereas PTMS can form three bonds, with many combinations of surface–PTMS and PTMS–PTMS bonds possible. The mechanism of growth of the silane film has previously been found to differ when using different substrates and silanes, with some substrates showing Langmuir-like growth and others showing an oscillation in the film growth [16,17]. It is therefore important to investigate how the silanes grow on a carbon surface in order to obtain the best silane coverage.

In order for a silane film to grow on a surface, the silane molecules (R’Si(OR)_3_) must first undergo hydrolysis to form reactive tri-silanol molecules (R‘Si(OH)_3_) [1,2]. These tri-silanols can then undergo condensation with each other or a hydroxylated surface, resulting in film growth for both PTMS and PDMMS, and oligomerisation of PTMS in solution [1,2]. It is therefore important that a carbon surface is oxidized before a silane film can be grown. The carbon surface used for this work is highly oriented pyrolytic graphite (HOPG), and was chosen due to its simple, well known structure, which again simplifies the system to aid in the study of the film growth mechanism. Conventionally, wet chemical methods are used to create the necessary oxide layer on the carbon surface. This paper demonstrates the use of oxygen plasma to grow an oxide layer, which is then further functionalized with a silane film. Some work has been done previously by Schade et al. [15] on functionalizing carbon/polymer materials with an oxygen layer prior to silane film deposition, however the plasma treatment conditions used were quite different and there was no quantitative analysis performed on the increase in oxygen content on the surface [15]. Previous work done by Taylor has shown how the atomic percentage of oxygen present on a HOPG surface changes with several plasma parameters, including carrier gas pressure, RF-coupling power, and treatment time [18]. The plasma conditions chosen for this work were based off this study. There are many advantages of plasma oxidation, including the very small amounts of oxygen gas used compared to the large amounts of solvents and chemicals in wet chemical methods, and the ease of controlling oxidation levels through modification of several plasma growth parameters.

**Figure 1:**
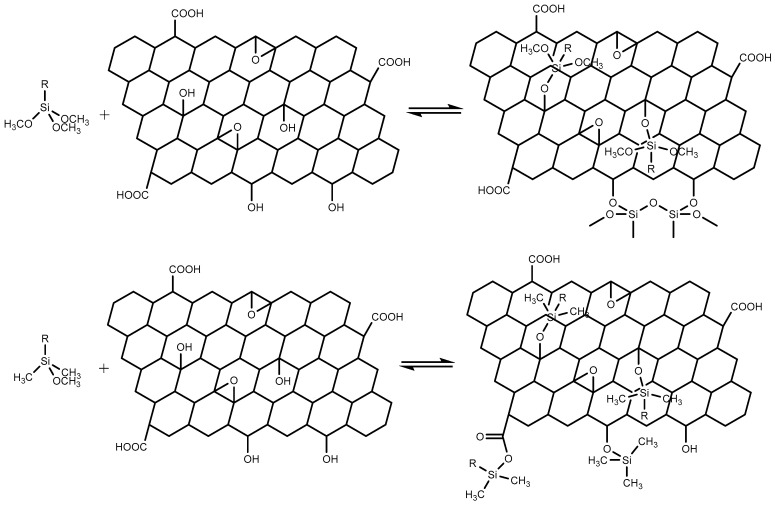
Reaction of (**top**) a tri-methoxy silane and (**bottom**) a mono-methoxy silane with an oxidized graphite surface.

The reaction of a silane with this oxidized HOPG surface is shown in Figure 1. The number of silane molecules that will bond to the HOPG surface will depend on the number of hydroxyl groups on the silane molecule and the concentration of possible bonding sites on the HOPG, and therefore on the plasma treatment parameters. It is desirable to increase the number of bonding sites to maximize silane film formation; however, this will also increase the disruption of the HOPG lattice. It is therefore necessary to find a balance between the number of bonding sites and the modification to the morphology and bonding of the surface layer of graphite.

Silane films on carbon materials have been examined previously using atomic force microscopy (AFM) [6,14], Fourier transform infrared spectroscopy (FTIR) [4,7,8,9,11,12,13], X-ray photoelectron spectroscopy (XPS) [7,8,9,13,14], transmission electron microscopy (TEM) [4,7,9,12,13], time-of-flight secondary ion mass spectroscopy (TOF-SIMS) [14], scanning electron microscopy (SEM) [8,9,14], X-ray diffraction (XRD) [8], Auger spectroscopy [15], and Raman spectroscopy [8,9]. The information from these previous studies can be combined to confirm the presence of the silane film and investigate the morphology and chemical composition of silanes on carbon materials. However, these factors have not all been investigated for one single system and thus detailed information about the film composition and morphology has not been obtained. In this investigation, silanised HOPG was characterized using XPS and scanning Auger microscopy (SAM). The use of SAM for characterization of this system is novel and has the advantage of providing detailed information on the chemical composition of the films formed with high spatial resolution and surface sensitivity, simultaneously showing nanoscale resolution SEM images. This allows for a more complete understanding of the system under study by providing additional information on the structure and morphology simultaneously.

## 2. Materials and Methods 

HOPG (12 mm × 12 mm × 2 mm, ZYB grade) was obtained from Coherent Scientific Australia, (Hilton, Australia) and prepared for treatment by cleaving with adhesive tape. The oxygen gas used to create oxygen plasma was high purity research grade (99.95% purity) from BOC. PTMS (97%) was obtained from Sigma Aldrich, Sydney, Australia, and PDMMS (97%) was obtained from Silar Laboratories, Riegelwood, NC, USA.

The oxygen plasma used in this research was produced using a radio-frequency (RF) inductively coupled antenna applying a power of 30 W to the carrier gas at a pressure of 1 × 10^−2^ Torr, with samples exposed to the plasma for 30 minutes.

XPS characterization was carried out using a Leybold-Heraeus LHS-10 system with an EA-10/100 concentric hemispherical analyzer (CHA) electron spectrometer and a SPECS XR-50 Mg Kα (1253.6 eV) X-ray source. A base pressure of ~1 × 10^−9^ Torr, take-off angle of 90°, and pass energy of 20 eV were used. SAM characterization was carried out using a PHI Model 710 Scanning Auger Nanoprobe, ULVAC-PHI, Chanhassen, MN, USA. A base pressure of ~1 × 10^−9^ Torr was achieved in the chamber, with characterization performed using a 10 kV 10 nA electron beam.

The sample area characterized with XPS is approximately 10 mm × 5 mm, which is most of the sample surface. XPS survey scans were taken over the binding energy range of 0–1200 eV with the spectrometer in constant retarding ratio mode. High resolution spectra were taken of the C1s peak over the range of 270−305 eV with the spectrometer in constant analyzer energy mode.

The sample area characterized with SAM depended on the features of interest, but generally ranged between 5 μm and 500 μm, which is a small section of the sample surface. As a result of this small sampling area, scans were acquired at several locations on each sample, resulting in a range of elemental concentration values. In-situ SEM images were taken of the samples in areas of interest, then Auger electron spectroscopy (AES) survey scans were taken in these areas with the kinetic energy range of 30−2030 eV. After the composition of the surface had been determined using the survey scans elemental maps of carbon, oxygen, and silicon were taken of areas of interest to show the uniformity of the silane film across the surface. Depth profiles were also taken in areas where the silane film was present to determine thickness. The beam energy used was either 10 kV 10 nA or 3 kV 5 nA, with a step size of 1.0 eV and a time per step of 10 ms for survey scans. Elemental maps were created using a 3-point acquisition method with a spatial resolution of 256 × 256 pixels and step size of 1.0 eV and time per step of 10 ms. Depth profiles were created using a window acquisition mode with step size of 1.0 eV, a time per step of 10 ms, and a sputter rate of 8.5 Å.min^−1^.

XPS data was processed using CasaXPS Version 2.3.16 (Casa Software Ltd), where fitted peak areas from survey scans were corrected using atomic sensitivity factors to determine the atomic concentrations of elements present on the sample surface. The high resolution C1s peaks were deconvoluted using CasaXPS, but no clear trend was observed so the results are not included here.

SAM data was processed using PHI’s MultiPak software, where peak areas from differentiated survey scans were corrected using atomic sensitivity factors to determine the atomic concentrations of elements present on the sample surface. MultiPak was also used to create red-green-blue (RGB) overlays of elemental maps and to determine atomic concentrations of elements in the depth profiles.

The various treatments applied are described in Table 1. Samples were prepared by drop-casting either neat silane (50 μL) or a drop-wise mixture of silane and Milli Q water (50:50, 50 μL total volume) to determine if the addition of water is necessary for film growth. For the 25 μL case, the total number of silane molecules introduced to the surface is estimated to be ~ 10^20^ and with a typical surface atomic density of ~10^15^ atoms per square centimeter exhibited by most materials, a multi-layer film is expected. The samples were left for three minutes and then either rinsed with excess water or dried with nitrogen before curing at 80 °C for two hours, before a final rinse to examine the impact of rinsing on the film pre-cure. The condensation reaction is an equilibrium process, thus the curing step is needed to force this reaction to completion. Some samples were also treated with PTMS without oxygen plasma to confirm the necessity of a hydroxylated surface. 

## 3. Results

### 3.1. PTMS

#### 3.1.1. XPS Results

The elemental compositions for both the PTMS and PDMMS samples as found by XPS are given in Table 2. Freshly cleaved HOPG contains around 1% oxygen, which is shown by XPS increasing to ~10–11% oxygen after the oxygen plasma treatment. XPS analysis of the C 1*s* peak of the oxidized HOPG surface showed the presence of carbonyl, ether, and hydroxyl and epoxy species in approximately equal amounts [18]. The different treatments all resulted in a different extent of silane film formation as shown by the varying silicon concentrations, with neat silane dropped on PTMS (PTMS 1) exhibiting the highest silicon content. However, it was thought that some of this silicon could be the result of silanes physisorbed to the surface rather than forming a covalently bonded film, thus the next sample was rinsed before curing (PTMS 2). This resulted in a significantly reduced silicon content (74.6% less than the unrinsed sample PTMS 1), but the silicon present was due to the bonded film and it is expected that most, if not all, of the physisorbed species had been removed. Water was also added with the PTMS for the next sample (PTMS 3) in an attempt to achieve better film growth, which resulted in a silicon content that was greater than the rinsed sample, but not as high as the neat PTMS (58.3% less than PTMS 1). The final variable tested for PTMS was the necessity of the oxygen plasma treatment. PTMS and water were dropped on freshly cleaved HOPG and either cured (PTMS 4) or rinsed and cured (PTMS 5). The cured sample had a silicon content that was almost as high as PTMS 1 (9.0% reduction from PTMS 1), however rinsing resulted in a much smaller silicon concentration (91.5% reduction from PTMS 1). This vast reduction with rinsing supports the conclusion that much of the silicon present in PTMS 1 is due to the physisorbed species rather than a covalently bonded film. 

#### 3.1.2. SAM Results

**Figure 2:**
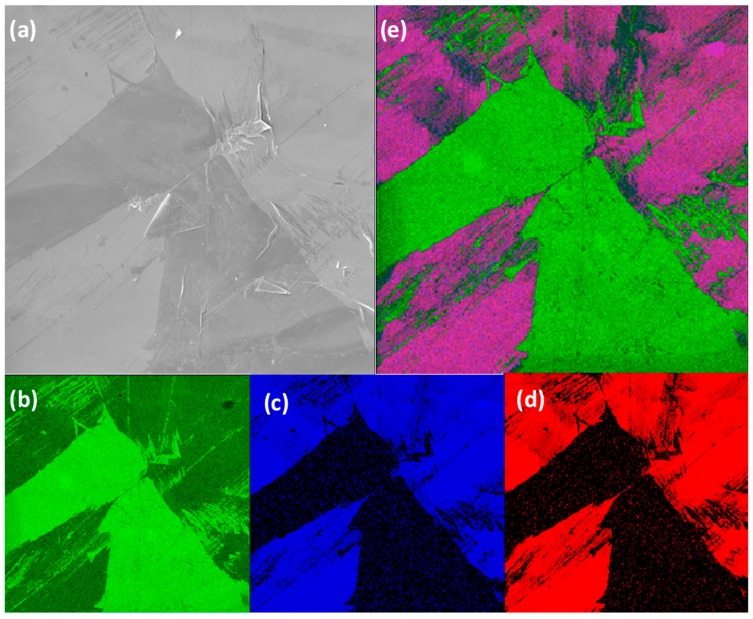
SEM image from propyltrimethoxysilane (PTMS) 3 (**a**), elemental maps of carbon (**b**), oxygen (**c**), and silicon (**d**), along with an RGB overlay of these three maps (**e**) (Field of view (FOV) 200 μm).

**Figure 3:**
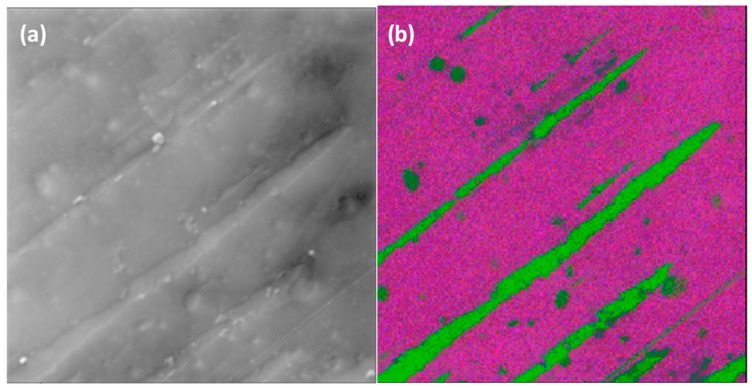
SEM image from propyltrimethoxysilane (PTMS) 3 (**a**,**b**) RGB overlay of carbon (green), oxygen (blue), and silicon (red) elemental maps (FOV 200 μm).

The elemental concentrations for the PTMS and PDMMS samples as found by SAM are given in Table 3. Freshly cleaved HOPG contains around 0.6–1.3% oxygen, while oxygen plasma treatment results in 9.9–12.9% oxygen. The elemental concentrations found with SAM are somewhat different to those found with XPS, with the differences being attributed to the sampling depth (up to 5 nm for SAM and up to 10 nm with XPS) and the sampling area (~2.5 × 10^−7^ m^2^ for SAM and ~1 × 10^−4^ m^2^ for XPS). It is thus expected that the silicon and oxygen concentrations will be larger and the carbon concentration smaller in SAM due to the greater surface sensitivity, and this is what was observed. Despite these differences in measured elemental concentrations, the same trends in changing concentrations are observed in SAM as in XPS, with PTMS 1 having the highest silicon content, followed by PTMS 3 then PTMS 2. The large ranges of values for silicon concentration indicate that the film is not consistent across the surface, which is also shown in elemental maps of Figure 2 and Figure 3.

SAM was also used to create elemental maps showing the distribution of carbon, oxygen, and silicon. Figure 2 shows the SEM of an area on PTMS 3, along with the carbon, oxygen, and silicon elemental maps, and an overlay of the three maps. There are two distinct regions in the SEM, and the chemical composition of these regions are different. The darker areas of the SEM image (Figure 2) have very little silicon or oxygen but a large amount of carbon, while the lighter areas contain a larger concentration of silicon and oxygen but less carbon. The same trend is observed in Figure 3, although the surface appears different between the two areas shown. It is expected that the thickness of the film will also vary across the surface due to the formation of PTMS oligomers. As PTMS can simultaneously form bonds with the surface and other PTMS molecules, the film can consist of single molecules bonded to the surface through all three possible sites, very large oligomers bonded to the surface only through one site, or anything between. This makes it difficult to form a consistent film across the entire surface, as there is a range of different oligomer structures formed from the PTMS, and therefore it is likely that each area of the surface will have a variety of oligomers attached to it that cannot be predicted. The addition of water to the PTMS treatment (PTMS 3) seems to encourage the bonding of PTMS molecules to each other rather than to the surface. Although the regions with the highest silicon concentration in PTMS 3 are greater than those in PTMS 2, there is a much larger range of silicon concentrations in PTMS 3 indicating that the coverage is much less uniform.

**Figure 4:**
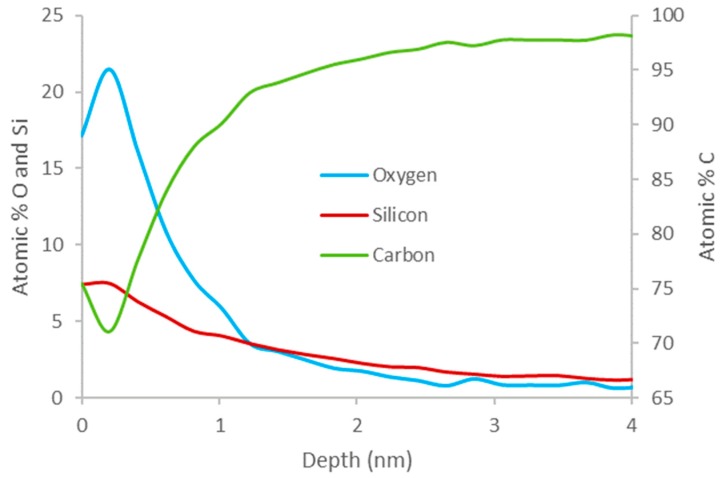
Depth profile of propyltrimethoxysilane (PTMS) 3.

Depth profiling was also performed on PTMS 2 and PTMS 3 to determine the thickness of the silane film. Figure 4 shows a depth profile indicating the change in concentration of carbon, oxygen, and silicon as a function of depth into the surface of PTMS 3. It can be seen that the initial concentration of silicon is 7.5%, which rises very slightly for ~0.2 nm before decreasing consistently down to 1% by 3 nm. The initial concentration of carbon is 75%, which decreases to 71% at a depth of ~0.2 nm, then increases to 98% by 3 nm. The oxygen profile varies inversely to carbon, with an increase in concentration from 17% to 22% at 0.2 nm, before decreasing to 1% by 3 nm. The thickness of the film is therefore 3 nm. The profile for PTMS 2 is a very similar shape, but the film is slightly thinner at ~2 nm.

### 3.2. PDMMS

The results from the PTMS investigation showed that oxygen plasma treatment of the HOPG was required before silane deposition to get a lasting film. Hence for the case of PDMMS, samples were not deemed worth making without this step.

#### 3.2.1. XPS Results

As can be seen in Table 2, neat PDMMS (PDMMS 1) produced far less silicon on the surface than was present in PTMS 1, which is likely due to the differences in structure and bonding abilities of the two silanes. Unlike the tri-methoxy silane, the mono-methoxy PDMMS can only make one covalent bond, either to the surface or another PDMMS molecule. This is expected to result in a more uniform film, as there are no large oligomers present to create thicker areas. When the sample was rinsed before curing (PDMMS 2), the silicon content was halved (48.3% reduction from PDMMS 1), and when water was added with the PDMMS (PDMMS 3), the silicon content was halved again (76.5% reduction from PDMMS 1). In the same manner as was described for PTMS, rinsing the sample before curing removes physisorbed species, so the decrease in silicon content is expected. Where PDMMS differs from PTMS is with the addition of water at the same time as the silane. While this resulted in an increase in silicon with PTMS, there was a decrease with PDMMS, which can be explained by differences in the structure of the two silanes. If the presence of water encourages bonding between silane molecules, then there will be fewer PDMMS molecules available to bond with the surface and therefore a thinner or less uniform film is created.

#### 3.2.2. SAM Results

The elemental concentrations for PDMMS in SAM (Table 3) follow the same trend as they did in the XPS measurements for PDMMS 1 and PDMMS 2, however no silicon was found using SAM in PDMMS 3. As discussed above, the amount of silane available to bond to the surface in PDMMS 3 is much less. There was a small amount of silicon observed for PDMMS 3 using XPS (1.9%), however there was no silicon observed in SAM. The difference between these numbers is most likely due to the different sampling areas of XPS and SAM. XPS takes an average over a very large area, so it will pick up small areas of silane film, however the areas examined through SEM images and AES spectra with SAM did not contain any of these small film sections.

Elemental maps of PDMMS 2 reveal a uniform covering of silicon, which agrees with the idea that PDMMS forms a monolayer as it cannot bond to other PDMMS molecules at the same time as it bonds to the surface. Maps of PDMMS 3 showed very little presence of silicon, in agreement with the elemental concentrations found using SAM.

Depth profiles of both PDMMS 2 and PDMMS 3 show a silane thickness of ~0.5 nm. Unlike the PTMS films, the PDMMS films show a rapid decrease in silicon concentration. PTMS 2 starts with ~4% silicon, which decreases to 1% by ~0.5 nm. The oxygen concentration was 11% initially, and decreased down to 1% by 0.75 nm, with the concentration of carbon increasing from 85% to 98% over the depth. PDMMS 3 shows the same pattern, but the initial silicon concentration was 1.5%. These thicknesses agree with the formation of a monolayer with no cross-linking between adsorbate molecules.

## 4. Discussion

The formation of a silane film on HOPG appears to be strongly dependent on the chemical structure of the particular silane used, thus the two silane cases studied in this work will be considered separately. PTMS has the ability to bond to both the surface and to other PTMS molecules via each of its three methoxy groups once they are hydrolysed to form silanols (Si–OH)_3_. This can lead to large oligomers that are bound to the surface by only a few covalent bonds, as well as single PTMS molecules bonded to the surface by substitution of all three methoxy groups with covalent bonds, and anything in between. These possible structures make it unlikely that there will be a complete, even film across the entire surface, as the distribution and structure of silane molecules and oligomers will not be uniform. The addition of water during PTMS deposition seems to encourage the preferential bonding of PTMS molecules to each other over bonding to the surface, as the coverage of silicon is less uniform in PTMS 3 than in PTMS 1, with some areas having almost no silicon and others exceeding 20%. These areas also appear different in SEM images, with darker areas containing little to no silicon and lighter areas containing large amounts of silicon. This is demonstrated in Figure 2, which shows that in darker areas of the SEM there is very little silicon and oxygen, while there is a large amount of carbon. The thickness of the silane film also varies across the surface. One area of PTMS 3 has a silane layer that is almost 4 nm thick (Figure 4), however this is not the same across the entire surface. The XPS results verify this. For non-hydrolysed silane molecules the carbon:oxygen ratio will be 6:3 (or 2:1), and for fully hydrolysed it would be 3:3 (or 1:1), whereas the observed ratio for samples PTMS 1 and PTMS 4 is ~3.3:1 due to the large cracks that occur upon curing. In order to obtain a covalently bonded film on a HOPG surface the results demonstrate that it is necessary to perform the oxygen plasma treatment first, and it is preferable to rinse the sample after curing to remove physisorbed species. Further studies on the addition of water to PTMS need to be undertaken to attempt to control the oligomerisation, however it has been observed that the addition of water encourages the formation of oligomers and should be avoided if a thinner, more uniform film is desired. 

Unlike the tri-methoxy silane PTMS, PDMMS contains only one hydrolysable methoxy group and therefore can bond only to either the surface or another PDMMS molecule to form a rather stable dimer in solution, resulting in a single uniform layer expected across the surface. The addition of water to the PDMMS results in many of the molecules bonding to each other rather than the surface, as shown by the decrease in silicon content on the surface by XPS and the very low amount present in SAM. This low concentration found with SAM suggests that there is non-uniform coverage of PDMMS across the sample, as there were very few areas where silicon was observed. It is thus suggested that to obtain a uniform film with the best film coverage water should not be added to the system with the silane, but rinsing should be performed after curing to remove physisorbed species.

## 5. Conclusions

PTMS and PDMMS films were successfully grown on oxidized HOPG, with plasma oxidation shown to be necessary for film formation. It is also necessary to rinse samples both before and after annealing to remove physisorbed silanes. However, the two silanes differ in their behavior with water presence. PTMS has better film formation when water is added with the silane while PDMMS films form best without addition of water. Additionally, PTMS forms thicker, more inconsistent films due to the presence of large oligomers, while PDMMS forms a thin film, more consistent with a monolayer of silane. Continuing work will focus on the ability to control silane adsorption by patterning the oxidation of the surface.

## Figures and Tables

**Table 1:** Treatment steps applied to each propyltrimethoxysilane (PTMS) and propyldimethylmethoxysilane (PDMMS) sample.

Sample	Oxygen Plasma	Water	Rinse before Curing	Rinse after Curing
PTMS 1	X			X
PTMS 2	X		X	X
PTMS 3	X	X	X	X
PTMS 4		X		X
PTMS 5		X	X	X
PDMMS 1	X			X
PDMMS 2	X		X	X
PDMMS 3	X	X	X	X

**Table 2:** Elemental composition for each propyltrimethoxysilane (PTMS) and propyldimethylmethoxysilane (PDMMS) sample found by XPS.

Sample	% C	% O	% Si
PTMS 1	56.3	17.7	26.0
PTMS 2	81.1	12.3	6.6
PTMS 3	77.4	11.8	10.8
PTMS 4	58.6	17.8	23.6
PTMS 5	94.0	3.8	2.2
PDMMS 1	82.0	9.9	8.1
PDMMS 2	87.4	8.4	4.2
PDMMS 3	92.2	5.9	1.9

**Table 3:** Elemental composition for each propyltrimethoxysilane (PTMS) and propyldimethylmethoxysilane (PDMMS) sample found by SAM.

Sample	% C	% O	% Si
PTMS 1	38.4–52.3	17.7–24.1	26.2–37.5
PTMS 2	58.8–28.9	15.9–19.2	11.3–18.2
PTMS 3	69.6–97.1	1.0–16.5	1.9–21.7
PTMS 4	44.7–57.9	19.3–28.8	22.8–29.8
PTMS 5	93.4–95.7	1.5–3.1	2.7–4.3
PDMMS 1	59.2–67.3	17.0–21.4	15.7–21.3
PDMMS 2	78.6–81.2	10.3–11.3	7.6–10.1
PDMMS 3	90.5–96.1	3.9–9.5	0
